# A new role for a tumor-suppressing protein

**DOI:** 10.7554/eLife.35111

**Published:** 2018-03-01

**Authors:** Jeremy S Setton, Simon N Powell

**Affiliations:** 1 Department of Radiation OncologyMemorial Sloan Kettering Cancer CenterNew YorkUnited States; 2 Molecular Biology ProgramMemorial Sloan Kettering Cancer CenterNew YorkUnited States

**Keywords:** p53, genome instability, replication fork, DNA replication, cancer, replication stress response, Human, Mouse

## Abstract

In addition to its role in preventing tumors, the protein p53 appears to participate in a DNA repair process known as the replication-stress response.

**Related research article** Roy S, Tomaszowski KH, Luzwick JW, Park S, Li J, Murphy M, Schlacher K. 2018. p53 suppresses mutagenic RAD52 and POLθ pathways by orchestrating DNA replication restart homeostasis. *eLife* **7**:e31723. doi: 10.7554/eLife.31723

Over half of all cancers involve mutations in a protein called p53. Dubbed the ‘guardian of the genome’, p53 can kill mutated cells or prevent such cells from multiplying, which stops tumors from growing. However, if p53 itself becomes faulty, cells with damaged DNA can accumulate and potentially lead to cancer. Besides its ability to eliminate ‘rogue’ cells, p53 may also be able to help prevent permanent mutations from appearing in the first place (Williams and Schumacher, 2016). For example, it can remove damaged bases and nucleotides from DNA, or promote mechanisms that repair harmful DNA breaks (Offer et al., 2001; Romanova et al., 2004; Wang et al., 1995).

There has been mounting evidence that p53 may also be involved in DNA replication, the error-prone process by which a cell makes a copy of its DNA before it divides. When DNA replicates, the double-helix unzips and forms Y- shaped structures called replication forks. If replication is disrupted, the forks may slow down and stall. This activates the ‘replication-stress response’, a mechanism that can recruit proteins to repair damaged DNA, restart the stalled fork, and ensure that replication carries on without mutations. Now, in *eLife*, Katharina Schlacher and colleagues at the UT MD Anderson Cancer Center and the Wistar Institute – including Sunetra Roy as first author – report that p53 may have a previously unknown role as a regulator of the replication-stress response (Roy et al., 2018).

In particular, they used a technique called DNA fiber assays to measure the number of stalled and restarted replication forks. They found that when p53 is defective, stalled replication forks could not restart properly. The role of p53 in restarting replication is different from its role in eliminating damaged cells, with certain p53 mutants being able to perform one role but not the other. This finding was similar to what has been described about the regulation of homologous recombination by p53 (Romanova et al., 2004; Willers et al., 2000), and future work will determine whether these two sets of observations are connected.

Roy et al. then examined whether p53 regulates the restart of stalled forks indirectly (via activation of gene expression) or directly (through protein-protein interactions at the replication fork). When a fork stalls, multiple molecules are recruited at the site, where they trigger the replication-stress response: p53 is known to bind with several of these (Byun et al., 2005; Romanova et al., 2004). Using normal and cancer cells from humans and mice, Roy et al. discovered that p53 is physically present at both active and stalled replication forks. When p53 worked correctly, it bound to the replication fork and ensured that replication resumed efficiently after it had passed any faulty regions of DNA. However, mutant p53 could no longer bind the replication fork, and stalled forks could not resume their activity properly.

Next, Roy et al. investigated how p53’s presence promoted stalled forks to restart. Their results showed that p53 recruited MLL3, a protein that can modify how chromatin – the structure into which the DNA is packed – is arranged (Zhu et al., 2015). These changes to the chromatin could attract another protein called MRE11 on the fork. This enzyme prevents DNA from breaking following replication stress (Berti and Vindigni, 2016; Costanzo et al., 2001; Ray Chaudhuri et al., 2016).

Finally, Roy et al. showed that when p53 was absent or mutated, two alternative DNA repair systems took over. These involved proteins called RAD52 and Polθ, which were increasingly recruited to the stalled replication forks. However, these two proteins are known to cause mutations, which could lead to an accumulation of genomic damage and perhaps cancer. Indeed, in breast tumors that lack a working version of p53, the DNA is damaged in ways that could have been provoked by RAD52 and Polθ.

Taken together, these results suggest that p53 is present at replication forks and plays a crucial role in the replication-stress response (Figure 1). When a fork stalls, p53 recruits proteins that fix the errors and efficiently restart replication. If p53 is absent or mutated, the stalled fork enlists other repair proteins that can make it restart, but which are prone to cause mutations. Are these mistakes enough to cause cancer? And could suppressing these back-up proteins help treat cancers caused by mutations in p53? This remains to be explored.

**Figure 1.**
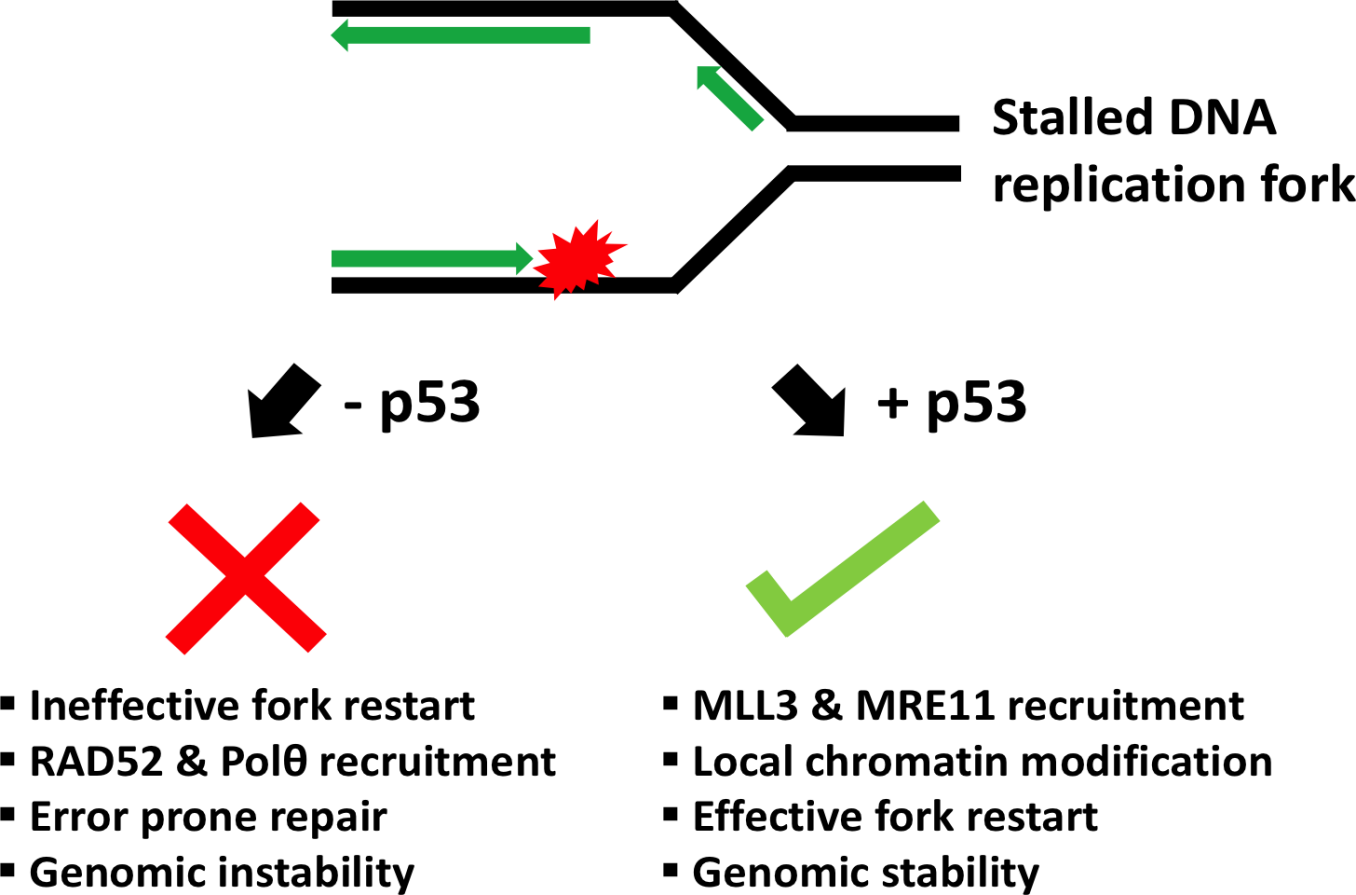
Schematic showing the role of p53 at the replication fork. When the DNA (black) replicates, the double helix opens to form a Y-shape called the replication fork. One new strand (the leading strand; green arrow) runs towards the replication fork, while the other (the lagging strand; green arrows at top) runs away from it. When a DNA lesion is encountered (in red), the replication fork may stall. Sometimes, however, the DNA continues to unwind ahead of the fork, leading to the formation of stretches of single-stranded DNA. This triggers a ‘replication-stress response’, which includes enlisting the protein p53 to the replication fork. In turn, p53 recruits MLL3 and then MRE11, which can modify the structure of nearby chromatin and prevent DNA breakage. This allows replication to restart and ensures that genetic information is preserved (genomic stability). Without p53, two proteins called RAD52 and Polθ are recruited to the stalled fork instead. Although these two proteins help to repair DNA, they can also lead to an accumulation of mutations (genomic instability).
